# Sulfadiazine—Chitosan Conjugates and Their Polyelectrolyte Complexes with Hyaluronate Destined to the Management of Burn Wounds

**DOI:** 10.3390/ma8010317

**Published:** 2015-01-16

**Authors:** Raluca Petronela Dumitriu, Lenuta Profire, Loredana Elena Nita, Oana Maria Dragostin, Nicolae Ghetu, Dragoș Pieptu, Cornelia Vasile

**Affiliations:** 1 “Petru Poni” Institute of Macromolecular Chemistry, Department of Physical Chemistry of Polymers, 41A Grigore Ghica Voda Alley, 700487 Iasi, Romania; E-Mails: rdumi@icmpp.ro (R.P.D.); lnazare@icmpp.ro (L.E.N.); 2 “Grigore T. Popa” University of Medicine and Pharmacy, Faculty of Pharmacy, Department of Pharmaceutical Chemistry, 16 University Street, 700115 Iasi, Romania; E-Mail: oana.dragostin@yahoo.com; 3 “Grigore T. Popa” University of Medicine and Pharmacy, Faculty of Medicine, Department of Plastic and Reconstructive Surgery, 16 University Street, 700115 Iasi, Romania; E-Mails: ghetu.nicolae@umfiasi.ro (N.G.); dragos.pieptu@umfiasi.ro (D.P.)

**Keywords:** chitosan, hyaluronic acid, sulfadiazine, polyelectrolyte complex, burn wound

## Abstract

In the present study polyelectrolyte complexes (PECs) based on new sulfadiazine-chitosan conjugates with sodium hyaluronate have been developed with potential use in treatment of burn wounds. The PECs were chemically characterized using Fourier Transform—Infrared Spectroscopy, Scanning Electon Microscopy and Near Infrared Chemical Imaging Technique. The swelling behavior and *in vitro* sulfadiazine release were also investigated. The antimicrobial activity was evaluated towards three bacterial strains: *Escherichia coli*, *Listeria monocytogenes* and *Salmonella thyphymurium*. The developed PECs demonstrated their antimicrobial efficiency against tested bacterial strains, the PECs containing sulfadiazine-modified chitosan being more active than PECs containing unmodified chitosan.

## 1. Introduction

Dressings are applied on open wounds to protect them from injuries and bacteria invasion. An ideal dressing should maintain a moist environment at the wound interface, allow gaseous exchange, act as a barrier to microorganisms, and remove excess exudates. It should also be non-toxic, non-allergenic, non-adherent and be easily removed without trauma; it should be made from a readily available biomaterial that requires minimal processing, possesses antimicrobial properties and promotes wound healing. Dressings made from natural or semisynthetic polymers are increasingly used to deliver drugs to acute, chronic and other types of wounds.

Chitin, chitosan, and their derivatives, prepared in various forms as hydrogels, nanofibers, membranes, micro/nanoparticles and sponges are promising biomaterials for wound dressing and other biomedical applications, such as drug and gene delivery, tissue engineering, *etc.* [1,2,3,4,5]. These have excellent properties being adhesive, biocompatible, biodegradable, nontoxic, hydrophilic, having also antimicrobial effect and oxygen permeability [6,7].

Chitosan stimulates cell proliferation and histoarchitectural tissue organization has hemostatic activity, which helps in natural blood clotting and blocks nerve endings, reducing pain. Chitosan gradually depolymerizes to release N-acetyl-beta-D-glucosamine, which initiates fibroblast proliferation, helps in ordered collagen deposition and stimulates increased levels of natural hyaluronic acid synthesis at the wound site. It also helps in faster wound healing and scar prevention [8]. Both chitin and chitosan fibers show good mechanical properties as mechanical strength of 1.5–2.5 g/dtex and elongation at break 8%–20%, being unique as raw materials for hi-tech bandages [9].

Hyaluronic acid (HA) exhibits an enhanced lubricating and water adsorption capacity influencing water retention and cellular events, such as attachment, migration and proliferation [10]. It has been used in ophthalmic surgery, arthritis treatment, in tissue engineering, as component of scaffolds for wound healing and implant devices [11]. The implants covered with HA and its derivatives reduce adsorption, adhesion and cellular proliferation of *Staphylococcus aureus* at least 100 times [12].

To further improve the chitosan (CS) properties and also to increase the ability as carriers for hydrophilic and lipophylic drugs, the chemical modification and copolymerization are applied, e.g., by reaction with thiazolidinone [13] or by grafting with poly(N-isopropylacrylamide), to obtain temperature-responsive hydrogels [14], by quaternization, grafting with alkyl aldehydes or alkyl ketones, to produce N-alkyl chitosan, with fatty acids, steroid derivatives, poly(ε-caprolactone) as was reviewed by Riva *et al.* [15,16,17]. CS-glutamate, CS-succinate, and CS-phthalate provide sustained release in basic medium [18]. There are also studies on chitosan conjugation with different drugs, such as CS-5-fluorouridine conjugate [19] or CS-alendronate conjugate [20]. CS-doxorubicin conjugation was carried out using succinic anhydride as a crosslinker. Trastuzumab was conjugated to self-assembled CS-doxorubicin conjugate (CS-DOX) nanoparticles (particle size, 200 nm) via thiolation of lysine residues and subsequent linking of the resulted thiols to chitosan. The monoclonal antibody, trastuzumab, was used as a targeting agent in nanoparticles carrying the antitumor drug, doxorubicin, specifically to its site of action [21]. The mucoadhesive property of CS, especially in an acidic (<pH 6.0) environment, was increased by conjugating an aromatic sulfonamide group at the C2-N position of chitosan. The CS-4-carboxybenzene sulfonamide conjugate showed antibacterial activity against *Escherichia coli* and *Staphylococcus aureus* [22,23].

Polyelectrolyte complexes (PECs) can be obtained by electrostatic interaction of amino groups C_2_ position in glucopyranosic units of CS with anionic groups (e.g., carboxyl) of polyanions of natural origin (such as pectine, alginate, carrageenan, xanthan gum, carboxymethyl cellulose, chondroitin sulfate, dextran sulfate, hyaluronic acid) or synthetic origin (e.g., polyacrylic acid, polyphosphoric acid, poly(L-lactide) [24]. PECs shows a very important sensibility at swelling, especially by pH modification, comparatively with covalently crosslinked hydrogels, which lead to the wide variety of applications [25]. PECs protects HA against enzymatic hydrolysis but only at pH values different of the optimum for enzymatic activity. PEC CS/HA as sponges and films allow the culture of various specific cells as keratocytes, which produces skin matrix accelerating wound healing after skin ablation without inflammatory reactions and toxicity for animals [26]. PEC obtained from chitosan and alginate can be applied to bandages or powders, which protect the wound, accelerates healing and prevent bacterial contamination [27]. Increase of the HA amount weakened the water vapor permeability, bovine albumin adsorption, and fibroblast adhesion, which are desirable characteristics for wound dressing. Comparatively with Vaseline, CS/HA films are more efficient in wound healing because they do not cause damage to the wound when the bandage is removed [28]. A wet treatment can be realized and both components of PEC contribute through their properties to the enhanced antimicrobial activity, and prevent wound damage during treatment. Polyelectrolyte complexation occurs under mild reaction conditions. PEC hydrogels exhibit a highly pH-sensitive swelling, therefore, they can be used for pH-controlled drug delivery in different conditions. The addition of HA to chitosans (chitosan hydrochloride and 5-methyl-pyrrolidinone chitosan) leads to a reduction in wound dressing hydration properties and a modulation of drug release [29].

Sulfonamides and their derivatives are among the most useful antimicrobial agents because of their low cost, low toxicity and excellent activity against bacterial infections [30]. Sulfadiazine (SDZ) is useful in the treatment of meningoccocal, staphyloccocal, streptococcal, *etc.* infections.

Based on this action, sulfadiazine was selected to modify CS and to obtain formulations with modified and retarded antimicrobial action. The aim of this study is to develop new polyelectrolyte complexes based on a new sulfadiazine-chitosan conjugate (SCS) with hyaluronic acid, as a new way to combine the bacteriostatic effect of chitosan with that of sulfadiazine, to control properties, drug release and also to improve the antimicrobial properties for wound healing application compared with CS/HA PECs in the treatment of the burn wounds.

## 2. Results and Discussion

### 2.1. Preparation of SCS/HA PEC Sponges

**Figure 1:**
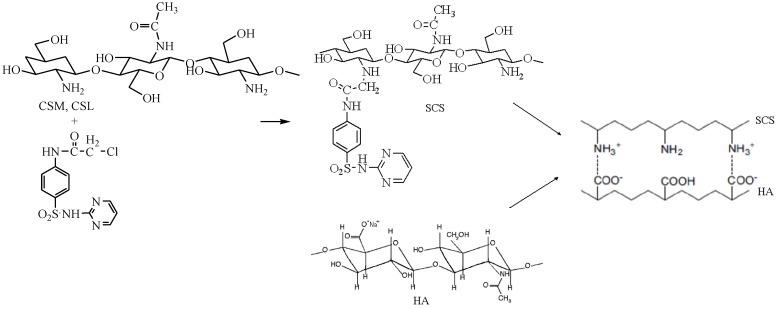
Formation of polyelectrolyte complexes (PECs) with sulfadiazine-chitosan conjugate (SCS) and sodium hyaluronate (HA).

PECs are formed based on the ionic interactions established between two oppositely charged polyelectrolytes, thus the pH value of the solution has to be controlled in order to ensure that both molecules are on the charged form. The suitable pH of the buffer solution for obtaining CS/HA and SCS/HA PECs depends on the pKa of the two polymers, namely the value of 6.5 reported for chitosan [31] and 2.9 for hyaluronate [32]. Selecting a pH between these two values, the requirements for obtaining PECs should be accomplished (Figure 1).

The pH of 4.12 was chosen for the acetate buffer solution used to obtain the PECs, due to the chitosan poor solubility or low reaction yields obtained at other pHs (3, 3.5, 4.5 and 5). For example, at pH ~3, a coacervate was formed as gel spheres but the dry product obtained resulted in a low reaction yield of ~40% showing that ionic interactions established between the two oppositely charged polyelectrolytes were not sufficient to accomplish PEC formation, because at this pH value the carboxylic groups of HA are still mostly protonated. Because of the low level of amino groups protonation, the chitosan with medium molecular weight (MMW, DD of 77.56%) and SCSM (DS of 31.36%) had both a poor solubility at pHs 5 and 4.5 because of a low level of amino groups protonation at this pH. SCS has a poor solubility due to the lower number of amino groups that can be protonated at acidic pH, being substituted with SDZ.

**Figure 2:**
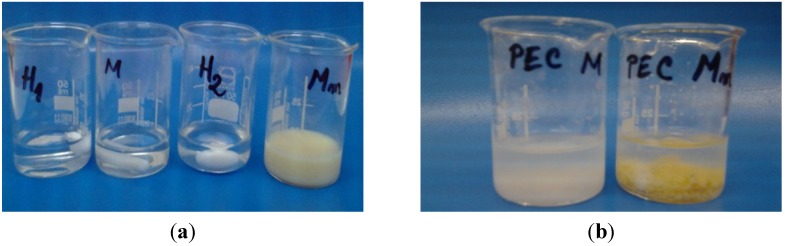
Images of the chitosan (CS) -M, sulfadiazine modified chitosan (SCS) -Mm and sodium hyaluronate (HA) -H_1_, H_2_ solutions before mixing (**a**) and after polyelectrolyte complexes (PECs) formation (**b**).

At pH 4.12, the CS and HA solutions were transparent and clear, while SCS (Mm) formed a turbid solution. In case of PECs prepared with chitosan the formation of a white precipitate was observed, while for PECs with sulfadiazine-modified chitosan (SCS) a light yellow precipitate was obtained (Figure 2). The pH of the solution increased slightly, at 4.62 for PEC M formation and at 4.44 for PEC Mm, which is a proof that various bonds are involved in ionic complex formation. The reaction yields obtained at pH 4 after freeze-drying of the samples are given in Table 1.

The aspect of the dried samples is like xerogels/aerogels for PEC L (chitosan low molecular weight—sodium haluronate) and PEC M (chitosan medium molecular weight—sodium hyaluronate), while PEC Lm (sulfadiazine modified chitosan low molecular weight—sodium hyaluronate) and PEC Mm (sulfadiazine modified chitosan medium molecular weight—sodium hyaluronate) are compact sponges. In the last case, it seems that in the case of sulfadiazine-modified chitosan complexes different kinds of specific interactions as ionic and hydrogen bonding are involved in complex formation therefore the PEC becomes more compact.

**Table 1:** Reaction yields obtained at pH 4.12.

PECs samples	Notation	Yield (%)
CSL:HA (1:1)	PEC L	69.7
CSM:HA (1:1)	PEC M	65.9
SCSL:HA (1:1)	PEC Lm	71.6
SCSM:HA (1:1)	PEC Mm	78.7

CSL: chitosan low molecular weight; CSM: chitosan medium molecular weight; SCSL: sulfadiazine modified chitosan low molecular weight; SCSM: sulfadiazine modified chitosan medium molecular weight; HA: sodium hyaluronate.

### 2.2. FT-IR Spectroscopy

**Figure 3:**
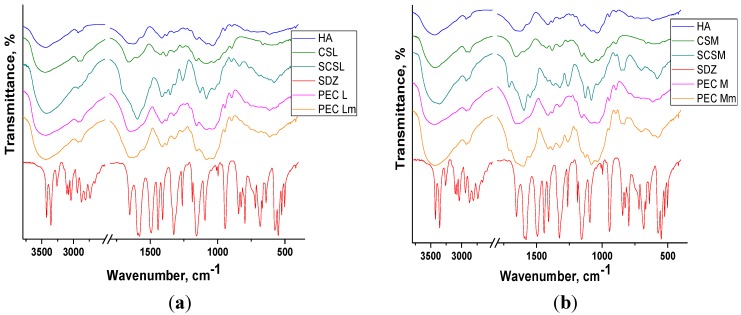
FT-IR spectra of components and of PEC sponges based on (**a**) CSL/SCSL and (**b**) CSM/SCSM (PEG: polyelectrolyte complexes, CSL/SCSL: chitosan low molecular weight/sulfadiazine modified chitosan low molecular weight, CSM/SCSM: chitosan medium molecular weight/sulfadiazine modified chitosan medium molecular weight).

The FT-IR spectra obtained for the PECs prepared and their components are presented in Figure 3a,b, in the spectral regions 2500–4000 cm^−1^ and 900–1900 cm^−1^, respectively. In the spectral region 2500–4000 cm^−1^ there are two absorption bands typically exhibited by polysaccharides: the broad band situated between 3360–3450 cm^−1^ assigned to the O–H stretching vibration (νOH) overlapped with the N-H stretching vibrations (νNH) and the band situated around 2921 cm^−1^ assigned to C–H stretching vibrations (νCH).

The broad band assigned to polysacharidic OH groups from chitosan is shifted to lower wavenumbers in the spectra of both the sulfadiazine-modified CS (SCS) and of PECs showing the presence of interactions between components.

The spectrum of sodium hyaluronate (HA) and of chitosan (CS) exhibit a band at 1626–1658 cm^−1^ attributed to C=O stretching vibrations (amide I) from the acetylated units with a shoulder at 1562–1594 cm^−1^ corresponding to N–H bend of amide II overlapped with the N–H bending vibration of the amine groups present in the desacetylated units of chitosan.

In the spectra of sulfadiazine-modified chitosan (SCS) and of PECs prepared with SCS (PEC Lm and PEC Mm) the bands corresponding to sulfadiazine (SDZ) can be identified: C–H band (para) between 700–895 cm^−1^ or the more intense bands assigned to C–N stretch (aryl) at 1260 cm^−1^ and to S=O stretch at 1082 cm^−1^ and 1130 cm^−1^ and some other bands are shifted to lower wavenumbers. In the spectra of PEC Mm the bands assigned to SDZ are more intense than in PEC Lm spectra.

In PECs spectra the bands corresponding to components can be identified, frequently overlapped due to similar structures of the two polysaccharides, chitosan and hyaluronate. In all FT-IR spectra of PECs the bands are wider and slightly shifted compared with the corresponding bands assigned to components, which indicates the presence of interactions leading to PECs formation.

### 2.3. Swelling Study

**Figure 4:**
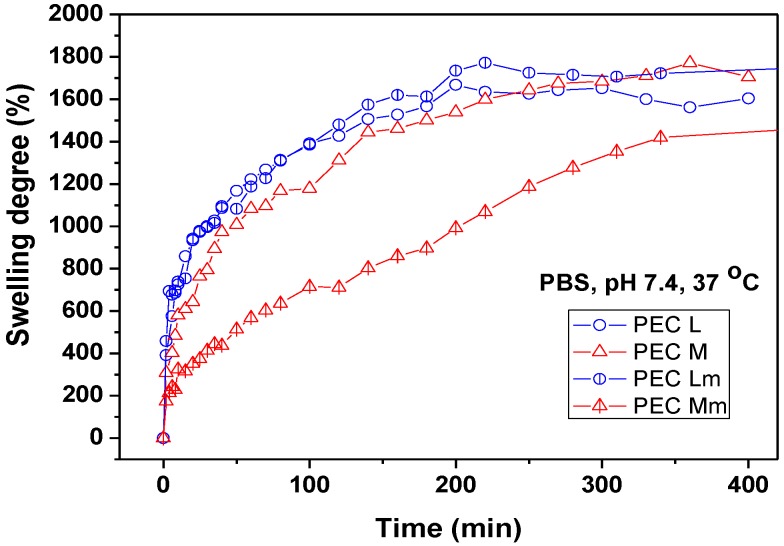
Swelling behavior of PEC (polyelectrolyte complexe) sponges in PBS (phosphate buffered saline) pH 7.4 at 37 °C.

The swelling measurements performed for PEC samples in PBS of pH 7.4 at 37 °C (Figure 4) showed the increased and faster swelling degree (SD) of PECs prepared with chitosan and PEC Lm up to ~1700% compared with PEC Mm, which reaches the maximum SD of ~1400% at a slower rate. The results obtained suggest that the prepared PEC sponges are quite hydrophilic, having an increased water uptake capacity. High swelling capacity is required for absorption of exudates and also to maintain a moist environment for good wound healing.

The swelling properties of the PECs can be explained based on the water-transfer mechanism and kinetics. The swelling kinetic parameters are summarized in Table 2.

**Table 2:** Swelling kinetic parameters of PEC sponges.

Sample	*n*	R^2^	k (min^n^)	R^2^
PEC L	0.33	0.999	18.76 × 10^−2^	0.998
PEC Lm	0.3	0.998	20.69 × 10^−2^	0.999
PEC M	0.36	0.999	13.45 × 10^−2^	0.999
PEC Mm	0.43	0.998	6.32 × 10^−2^	0.998

PEC L (chitosan low molecular weight-sodium haluronate); PEC Lm (sulfadiazine modified chitosan low molecular weight-sodium hyaluronate); PEC M (chitosan medium molecular weight-sodium hyaluronate), PEC Mm (sulfadiazine modified chitosan medium molecular weight-sodium hyaluronate).

The diffusion exponent (*n*) values suggest that the swelling mechanism of PEC sponges takes place generally by an anomalous transport, which occurs by coupling *Fickian* diffusion with the relaxation of the hydrogel network. Exception is sample PEC Mm, which has a diffusion coefficient closer to 0.5 (*n* = 0.43), indicating the approach to a *Fickian* diffusion mechanism. PEC Mm presents the smoothest and slowest swelling profile among the investigated PECs, because of the lowest swelling rate constant value, of 6.32 × 10^−2^ min^−0.43^, compared with the other three PECs investigated. This behavior upon swelling correlates with the slower release rate of the drug from this polymeric matrix.

The swelling behavior studies performed at pH 7.4 showed a lower stability of the PECs obtained with SCS compared with PECs prepared with neat CS. Both PEC Lm and PEC Mm maintained compact for weight measurements only up to 300–350 min at pH 7.4. Taking into consideration the results and observations from the swelling study, the drug release experiment was performed at acidic pH in order to be able to observe the prolonged SDZ release in time from SCS-based PECs.

### 2.4. Scanning Electron Microscopy (SEM)

**Figure 5:**
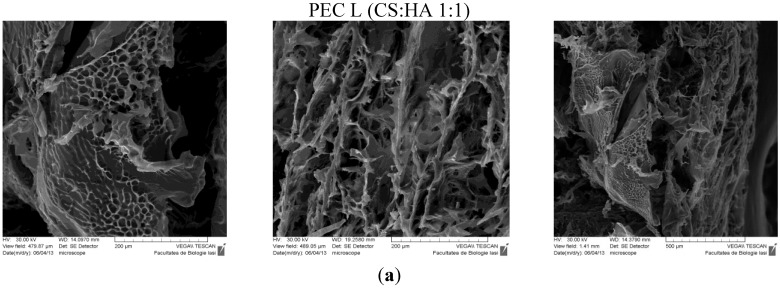

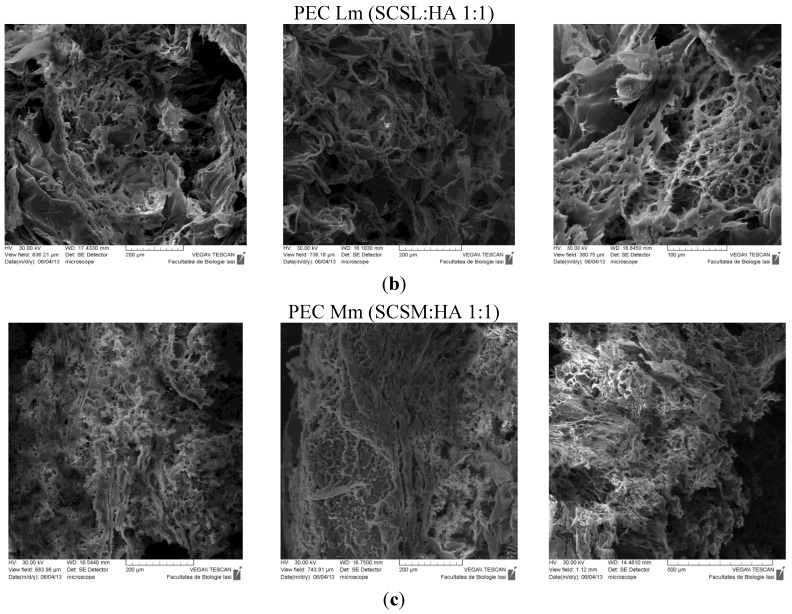
SEM images of PECs (polyelectrolyte complexes): (**a**) PEC L (chitosan low molecular weight-sodium hyaluronate); (**b**) PEC Lm (sulfadiazine modified chitosan low molecular weight-sodium hyaluronate); and (**c**) PEC Mm (sulfadiazine modified chitosan medium molecular weight-sodium hyaluronate).

The SEM technique allowed observation of the morphologic details of the PEC sponges both on the surface and on the edge/in fracture. In Figure 5 are presented the images obtained, showing a heterogeneous porous morphology with pores highly interconnected.

The sample PEC Mm seams to have an enhanced and more dense/compact porosity compared with the other samples, which allows an efficient entrapment of the drug within the 3D structure followed by a subsequent slower release.

### 2.5. Near Infrared Chemical Imaging (NIR-CI)

The NIR-CI method of analysis provides information about the spatial distribution of the components with the possibility to determine the degree of the chemical and/or physical heterogenity [33]. The evaluation of the components distribution in samples was made by near infrared chemical imaging (NIR-CI) using chemometric analysis method.

Based on multivariate analysis techniques and principal component analysis (PCA), partial least squares regression (Partial Least Squares-Discriminant Analysis, PLS-DA) and soft independent modeling of class analogy (SIMCA), the quantitative and qualitative information was extracted from the NIR spectral variables data cube.

**Figure 6:**
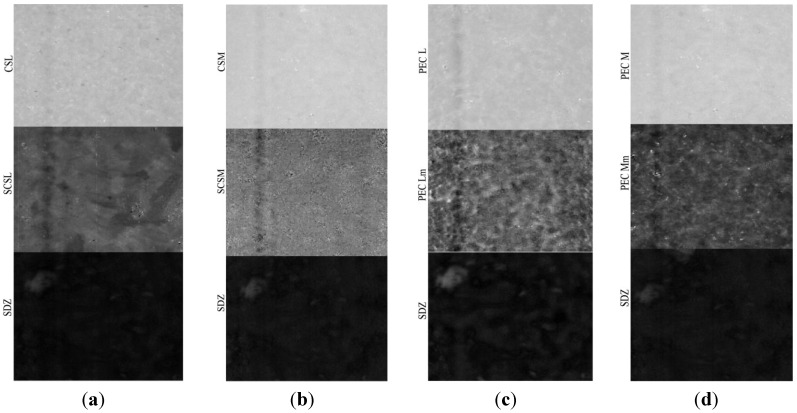
Partial Least Squares-Discriminant Analysis (PLS-DA) model for sulfadiazine (SDZ)-containing systems: (**a**) CSL-SCSL-SDZ (chitosan low molecular weight-sulfadiazine modified chitosan low molecular weight-sulfadiazine); (**b**) CSM-SCSM-SDZ (chitosan medium molecular weight-sulfadiazine modified chitosan medium molecular weight-sulfadiazine); (**c**) PEC L-PEC Lm-SDZ (chitosan low molecular weight:sodium hyaluronate-sulfadiazine modified chitosan low molecular weight:sodium hyaluronate-sulfadiazine); (**d**) PEC M-PEC Mm-SDZ (chitosan medium molecular weight:sodium hyaluronate-sulf adiazine modified chitosan medium molecular weight:sodium hyaluronate-sulfadiazine).

The PLS-DA models corresponding to the components (sulfadiazine-SDZ, sulfadiazine-modified chitosan and to PEC samples are depicted in Figure 6. It can be noticed that both the sulfadiazine-modified chitosan samples (SCSL and SCSM) and the PECs obtained (PEC Lm and PEC Mm) have a good homogeneity, but samples SCSM and PEC Mm have a visibly higher homogeneity degree, which corresponds to a more uniform drug distribution within the matrix; data that correlates well with the drug release behavior.

The prediction for these samples as non-classified (100% new compounds both as PEC and SDZ incorporated PECs noted as PEC L-SDZ, PEC Lm-SDZ and PEC M-SDZ, PEC Mm-SDZ, respectively, for low and medium average molecular weight unmodified and modified chitosan) shows that the conjugate with the SDZ was successfully obtained and the PECs were prepared as new and efficient drug release systems.

**Figure 7:**
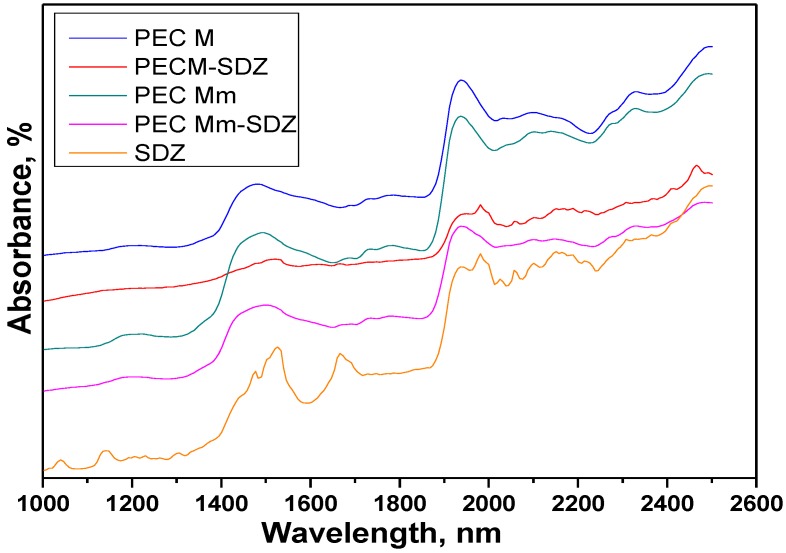
Compared Near-IR reflectance spectra of the polyelectrolyte complexe (PEC) sponges prepared without added sulfadiazine (SDZ) and of SDZ-loaded PECs (PEC M and PEC Mm).

The Near infrared compared spectra of the PEC hydrogel sponges, sulfadiazine (SDZ) and drug-loaded PECs with an air background are presented in Figure 7. Intense absorption bands corresponding to the functional groups of the components are observed in the full range of the spectra (Table 3).

**Table 3:** NIR-CI spectra bands (nm) assignment [34,35].

SDZ	PEC M	PEC M-SDZ	PEC Mm	PEC Mm-SDZ	Bands Assignment
1142, 1668, 1692 (shoulder)					C–H second overtone; C–H stretch first overtone
1475,1525	1475	1524	1490	1502	NH stretch first overtone, NH–CO groups (CO–NHR and RNH_2_ groups)
	1938 (strong band)		1938 (strong band)	1940 (wider band)	O–H bend second overtone, C=O stretch second overtone
1938, 1982, 2000 (shoulder)		1938, 1982, 2000 (shoulder)			N–H stretch/N–H in plane bend C–N stretch combination, NH bend combination
	2032		2036	2034 (very weak band)	C=O stretch second overtone
2057		2059			N–H combinations, N–H in plane bend C–N stretch
2096, 2150	2096	2096, 2152	2098	2098	C–H combination, R–NH_2_ overlapped O–H bend/C–O stretch combination
	2170		2145	2155	C–H deformation combination, C=O stretch combination
	2275		2273	2273	O–H stretch/C=O stretch combination
	2326		2326	2328	C–H stretch

SDZ—sulfadiazine; PEC M—chitosan medium molecular weight:sodium hyaluronate; PEC M-SDZ—chitosan medium molecular weight: sodium hyaluronate: sulfadiazine; PEC Mn—sulfadiazine modified chitosan medium molecular weight:sodium hyaluronate; PEC Mn-SDZ—sulfadiazine modified chitosan medium molecular weight:sodium hyaluronate: sulfadiazine.

Sulfadiazine (SDZ) near-IR spectra evidenced the bands at 1142 nm corresponding to C–H second overtone and the band at 1668 nm with a shoulder at 1692 nm corresponding to C–H stretch first overtone, which can be attributed to ArCH from benzene ring and pyrimidine ring, respectively. Specific for SDZ are the bands at 1475 nm and 1525 nm attributed to N–H stretch first overtone (CONHR and RNH_2_ groups) and the weak band at 1938 nm coupled with the sharp band at 1982 nm with a shoulder at 2000 nm for N–H stretch/N–H in plane bend C–N stretch combination corresponding to NH_2_ group and –NH bonded to pyrimidine ring.

In the spectra of PEC M (CHTM: HA 1:1), a broad band appears at 1475 nm corresponding to NH stretch first overtone assigned to amino/NH_2_ groups of chitosan and amide/NH–CO groups from both chitosan and hyaluronate and an intense/strong band at 1938 nm corresponding probably to both O–H bend second overtone (polysaccharidic OH groups) and to C=O stretch second overtone (from both COO^−^ or amide NH-CO groups).

The spectra recorded for PEC Mm and PEC Mm-SDZ are quite similar with that of PEC M, but the broad band recorded at 1475 nm for PEC M is shifted to 1490 nm for PEC Mm and to 1502 nm for PEC Mm-SDZ and changes slightly the aspect for the last one showing the influence of the CONHR and RNH_2_ groups from SDZ with the peaks recorded at 1475 nm and 1525 nm. For PEC M-SDZ this band is shifted to 1524 nm and has a significantly modified aspect and decreased intensity.

The intense/strong band attributed to polysaccharidic OH groups at 1938 nm in PEC M spectra is shifted to 1940 nm and becomes larger and less intense in the spectra of PEC Mm-SDZ, being overlapped with the intense bands from SDZ corresponding to NH combinations. These particular bands and the ones recorded at 2057 nm and to 2096 nm, 2150 nm (corresponding to N–H in plane bend C–N stretch and to C–H combination) can be easily identified in the spectra of PEC M-SDZ at almost the same wavelengths as in SDZ spectra.

Meanwhile the spectra of PEC M, PEC Mm and PEC Mm-SDZ present larger bands, with lower intensity in the range 2000–2040 nm. The band at 2032 nm from PEC M spectra attributed to C=O stretch second overtone is slightly shifted to 2036 nm in the PEC Mm spectra and almost cannot be identified in the spectra of PEC Mm-SDZ which suggests the interactions between COO^−^ groups from HA with NH_2_ groups of SDZ. The bands at 2096 nm and 2170 nm from PEC M spectra are slightly shifted in PEC Mm spectra, but became larger, with lower intensity and shifted at 2098 nm and 2155 nm, respectively, in PEC Mm-SDZ spectra, demonstrating the presence of additional SDZ in sample.

These findings show that SDZ bands can be identified in the spectra of the physically loaded PEC sample, while the spectra of the PEC prepared with SDZ-modified CS (PEC Mm) was quite similar with that of PEC M, with larger bands such as PECs present generally, demonstrating that SDZ was well-bonded in the SDZ-CS conjugate as the NIR predictions identified the PEC obtained as a new compound.

The presence of mainly broad bands in the spectra of the PECs obtained suggests the presence of the ionic interactions between the two oppositely charged polyelectrolytes, chitosan and hyaluronate, leading to the formation of PECs.

### 2.6. Drug Release Kinetic Studies

**Figure 8:**
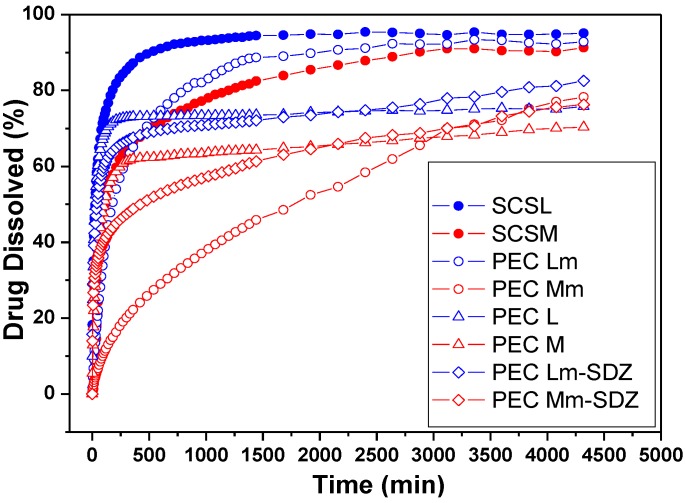
Sulfadiazine release profiles from chitosan (CS) conjugates (SCSL—sulfadiazine modified chitosan low molecular weight and SCSM—sulfadiazine modified chitosan medium molecular weight) and from SDZ-loaded PECs.

The *in vitro* sulfadiazine release was investigated at 37 °C, in acidic solution of pH 2.7. The drug release profiles are depicted in Figure 8. The sulfadiazine release kinetic parameters were calculated (Table 4).

The acidic pH of 2.7 was chosen for the drug release study in order to be able to observe the prolonged SDZ release from PECs based on SCS, which demonstrated a reduced stability at pH 7.4 compared with CS-based PECs during swelling studies.

The release rate and the amount of sulfadiazine released depends on the type of sample, either it is a conjugate or a PEC. Also the stability of the PECs in the dissolution medium is affected by their composition.

**Table 4:** Kinetic parameters for sulfadiazine (SDZ) release.

Sample	*n*	R	k·(min^n^)	R
PEC L-SDZ	0.36	0.997	3.58 × 10^−2^	0.999
PEC Lm	0.32	0.997	1.60 × 10^−2^	0.999
PEC Lm-SDZ	0.24	0.999	4.34 × 10^−2^	0.999
SCSL	0.30	0.999	3.53 × 10^−2^	0.999
PEC M-SDZ	0.33	0.999	2.55 × 10^−2^	0.999
PEC Mm	0.23	0.999	7.45 × 10^−3^	0.999
PEC Mm-SDZ	0.18	0.999	3.83 × 10^−2^	0.999
SCSM	1.00	0.991	8.94 × 10^−4^	0.998

PEC L-SDZ: chitosan low molecular weight:sodium hyaluronate:sulfadiazine; PEC Lm: sulfadiazine modified chitosan low molecular weight-sodium hyaluronate; PEC Lm-SDZ: sulfadiazine modified chitosan low molecular weight-sodium hyaluronate: sulfadiazine; SCSL: sulfadiazine modified chitosan low molecular weight, PEC M-SDZ: chitosan medium molecular weight:sodium hyaluronate: sulfadiazine; PEC Mm: sulfadiazine modified chitosan medium molecular weight-sodium hyaluronate; PEC Mm-SDZ: sulfadiazine modified chitosan medium molecular weight-sodium hyaluronate: sulfadiazine; SCSM: sulfadiazine modified chitosan medium molecular weight.

Previous studies performed on chitosan polyelectrolyte complexes and in particular PECs with hyaluronate have shown that complex stability is closely related with the chitosan DA (degree of acetylation), by decreasing when DA increases, the distance between charged groups (NH_3_^+^) increases. Thus the complex is less cooperative for large DA [36].

In our investigations the PECs obtained with SCS were more stable at acidic pH compared with the PECs obtained with neat CS, which disintegrate at pH 2.7 due to chitosan solubility by protonation of amino groups. SCSM has a DD (degree of desacetylation) of 77.56% (DA of 22.44%) so there was enough charge density at acidic pH to form a PEC with HA, but due to the increased DS of 31.36% a significantly lower number of NH_2_ groups were available for protonation in acidic media to induce solubility compared with CS or SCSL (DS of 16.66%). Thus by substitution with SDZ the PECs prepared with SCS became more stable at pH 2.7.

The SDZ release results showed a slower release rate from all samples containing CSM, either SCSM or PEC Mm, compared with samples containing CSL/SCSL. Also the release of up to 80% of SDZ in 72 h was recorded from almost all PEC samples, except PEC Lm, which has a similar behavior with SCSL. These findings demonstrate that the drug was well entrapped in the polymer matrix, facilitating its slower release.

For the PEC samples prepared with SCSM the release profiles are visibly smoother compared with the other samples, showing the slowest release rate and the lowest release percent during 50 h/~2 days. In particular the PEC Mm samples had a significantly slower release rate compared with PEC M and SCSM.

The slowest release rate was recoded for PEC Mm sample. The enhanced chains length/entanglement in CSM is favorable for keeping the drug inside the polymer matrix for longer time and releasing it slowly subsequently. Additionally, the presence of interchain hydrogen bonding might be also responsible for a better entrapment of the drug within the polymeric matrix and its slower release.

The values obtained for the diffusion exponent (*n*) at pH 2.7 correspond to an anomalous, non-Fickian mechanism for all samples, except sample SCSM (*n* = 1) when a case II transport mechanism is involved with zero order kinetics.

The decrease of the release rate constant values (k) for all SCSM-containing samples corresponds to a slower release rate and indicates a significantly prolonged delivery from samples containing CSM and especially SCSM.

The release studies were performed at pH 2.7 were all PECs show the approximately the same resistance and aspect. PECs of both modified chitosans are stable over a wide range of pH from pH 2.7 to 7.4 as also other authors reported [37] while PECs containing SDZ chitosan although they disintegrate at pH 7.4 still remain non-soluble. Therefore both types of PECs matrices could be useful for wound dressing where pH is higher than 4.

### 2.7. Antimicrobial Tests

The antimicrobial activity of the PECs obtained with CS, with SDZ-modified CS and with added SDZ to the PECs prepared was studied comparatively on three bacterial strains: *Escherichia coli*, *Listeria monocytogenes* and *Salmonella thyphymurium*. The results obtained are presented in Table 5.

**Table 5:** Antimicrobial tests results.

Sample	Inhibition (%)					
	*Escherichia coli*		*Listeria monocytogenes*		*Salmonella thyphymurium*	
	24 h	48 h	24 h	48 h	24 h	48 h
PEC L	29	38	58	89	17	43
PEC M	49	67	66	83	39	52
PEC Lm	59	63	77	100	100	100
PEC Mm	52	79	64	92	31	81
PEC L-SDZ	49	71	45	77	25	50
PEC M-SDZ	68	90	74	100	87	100
PEC Lm-SDZ	83	95	100	100	94	100
PEC Mm-SDZ	72	94	70	94	96	88

PEC L: chitosan low molecular weight:sodium haluronate, PEC M: chitosan medium molecular weight:sodium haluronate, PEC Lm: sulfadiazine modified chitosan low molecular weight-sodium hyaluronate, PEC Mm: sulfadiazine modified chitosan medium molecular weight-sodium hyaluronate, PEC L-SDZ: chitosan low molecular weight:sodium haluronate:sulfadiazine, PEC M-SDZ: chitosan medium molecular weight:sodium haluronate:sulfadiazine, PEC Lm-SDZ: sulfadiazine modified chitosan medium molecular weight-sodium hyaluronate-sulfadiazine, PEC Mm-SDZ: sulfadiazine modified chitosan medium molecular weight-sodium hyaluronate: sulfadiazine.

**Figure 9:**
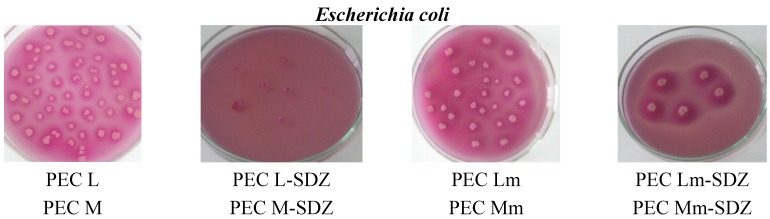

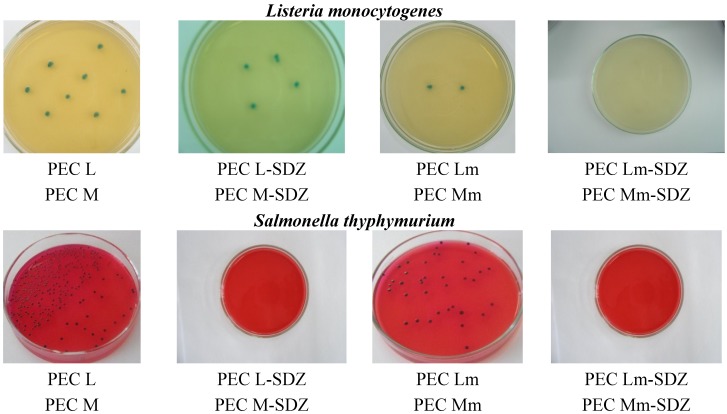
Representative microscopical aspects of the colonies of *Escherichia coli*, *Listeria monocytogenes* and *Salmonella thyphymurium* grown over polyelectrolyte complexes based chitosan/sodium hyaluronate (CS/HA PECs) without and with sulfadiazine (SDZ) modification.

The results obtained confirm the antimicrobial effect of CS-containing PECs ranging from 17% to 67% while SDZ has an inhibitory capacity of 70%–78%, while the SDZ/CS-containing samples have a significantly enhanced efficiency in activity, reaching even 100% inhibition of bacteria growth—mostly for samples with added SDZ proving their synergistic effect. For all three bacterial strains investigated, PECs Lm and Mm have an increased inhibition effect compared with CS-based PECs (PEC L and PEC M), while for the PECs with added SDZ, in particular PEC Lm-SDZ and PEC Mm-SDZ, the inhibition percent is even higher. Also the increase of antimicrobial activity was observed for PEC L and PEC M when SDZ was added (Figure 9). No differences are found in antimicrobial activity of the PECs containing CS with low and medium average molecular weight.

## 3. Experimental Section

### 3.1. Materials

Low (CSL) and medium (CSM) molecular weight chitosan were purchased from Sigma-Aldrich and sodium hyaluronate (HA) from *Streptococcus equis* was obtained from Fluka. Chitosan (CSL), a product of low molecular weight has a viscosity of a 5 wt% concentration solution of 20.000 cP, in 1% acetic acid (25 °C) and deacetylation degree of 77.8%. Chitosan (CSM), a product of medium molecular weight (Mw) of 190–310 kDa was purchased from Sigma-Aldrich. It has a viscosity of 200.000 cP, in 1% acetic acid (25 °C) and deacetylation degree of 78.5%.

Sulfadiazine (SDZ), a sulfonamide drug, was used for chitosan modification. The sulfonamides are synthetic bacteriostatic antibiotics with a wide spectrum against most gram-positive and many gram-negative microorganisms. Sulfadiazine is one of the short-acting sulfonamides used for the treatment of rheumatic fever and meningococcal meningitis. Sulfadiazine can be easily identified, being readily absorbed after oral administration and subsequently excreted largely in the urine [38]. As silver salt sulfadiazine is used locally to prevent and treat wound infections in patients with serious burns [39].

Sulfadiazine, also called sulfapyrimidine or 4-amino-N-(pyrimidin-2-yl)-benzen-sulfonamide is a white or white-yellow powder, sensitive to light with a melting point of 252–256 °C, insoluble in water, alcohol, ether, chloroform, soluble in acetone and diluted acidic or alkaline solutions [40]. By using sulfadiazine it was expected to increase the antimicrobial effect of the PECs obtained using SDZ-modified CS (SCS). The procedure of synthesis of the CS derivative with sulfadiazine is described in other paper [41].

### 3.2. Preparation of SDZ-Modified CS

The sulfadiazine-modified chitosan derivatives have been synthesized by reaction of chitosan medium molecular weight (CSM) and chitosan low molecular weight (CSL) with N-chloracethyl sulfadiazine using similar methods from the literature applied to other chitosan derivatives [42,43,44]. To a stirred solution of chitosan (CSM, CSL) (0.011 M) in 1% acetic acid (100 mL) a solution of N-chloracethyl sulfadiazine (0.0132 mol) in DMFA (50 mL) was added. The reaction mixture was stirred for about 24 h at room temperature and then the pH was corrected at 9 with 15% NaOH solution when the solid product precipitated. The products were washed five times with water, until the pH of the filtrate was 7. The final compounds were purified by dialysis against deionized water for 5 days and then freeze-dried on Alpha 1–2 LD Plus lyophiliser (Germany). The degree of substitution of this derivative was 31.64%. Both ionic and covalent bonds could be formed between CS and SDZ as can be remarked from the Figure 1.

### 3.3. Preparation of SCS/HA PEC Sponges

The SCS/HA and CS/HA PECs have been prepared by mixing the two polysaccharide solutions in 1:1 mass ratio, followed by freeze-drying of the insoluble polyelectrolyte complexes formed. Therefore 1 wt% solutions of HA and CS, respectively, SCS were prepared separately by dissolving 0.1 g of the polymer in 10 mL of a 0.1 M acetate buffer (pH = 4.12) solution. The two solutions were then mixed at room temperature in order to prepare the PECs: the CS/SCS solution was kept under continuous stirring (700 rpm) while the HA solution was dropped slowly into the chitosan solution and the mixture was left under vigorous stirring (1000 rpm) for 30 min. The formed PECs were isolated by centrifugation at 5000 rpm for 5 min, washed repeatedly with twice distilled water then were frozen and finally freeze dried overnight. UV-VIS spectra certified the purity of the systems.

### 3.4. Investigation Methods

#### 3.4.1. FT-IR Spectroscopy

Fourier Transform-Infrared (FT-IR) spectroscopy analysis was performed on HA, CS, SCS, SDZ and PECs in the range of 4000–500 cm^−1^, using a Bruker VERTEX 70 spectrometer (Billerica, MA, USA) in Transmittance mode. The dried formulations were grounded to powder, mixed with KBr, compressed into a tablet and then spectra were recorded at 4 cm^−1^ resolution.

#### 3.4.2. Swelling Behavior

The swelling behavior was studied by immersing dried pre-weight pieces of the prepared PEC sponges in a PBS buffer solution of pH 7.4, at 37 °C and measuring their swollen weight at predetermined time intervals. The swelling degree (SD) was evaluated using Equation (1) [45]:

(1) $$SD(\%)=\frac{\left(m-m_{0}\right)}{m_{0}}\times 100$$

where *m*_0_ is the mass of dry sample and *m* is the mass at moment “t” of swelling.

#### 3.4.3. Scanning Electron Microscopy (SEM)

The morphology of SCS/HA PEC sponges was analyzed by SEM. Samples were placed on a double-sided graphite tape, attached onto a metal support and coated with gold on a sputtering coater. Observations were performed with a VEGA II TESCAN scanning electron microscope. Magnification is indicated on pictures.

#### 3.4.4. Near Infrared Chemical Imaging (NIR-CI) Technique

NIR spectra were recorded on a SPECIM’S Ltd. Sisu-CHEMA, Finland controlled with Evince software package for processing the original image data. The system includes a Chemical Imaging Workstation for 1000 to 2500 nm NIR domains. The original image for each sample was taken with a NIR model spectral camera, respectively, an imaging spectrograph type ImSpector N17E with 320 and 640 pixel spatial resolution at a rate of 60 to 350 Hz. Each sample was scanned between 1000 and 2500 nm.

The NIR-CI data were collected on a SisuCHEMA (Finland) device, which employs SPECIM’s hyperspectral imaging technology on full NIR (1000–2500 nm) range. The system is equipped with a spectral camera, 320 × 640 pixel spatial resolution for a rate of 60–350 Hz. The hyperspectral camera has a mercury cadmium telluride (MCT) detector. The EVINCE image processing software was used for data analysis.

#### 3.4.5. *In Vitro* Drug Release Study

The *in vitro* release studies for sulfadiazine (SDZ) have been performed using a 708-DS Dissolution Aparatus coupled with a Cary 60 UV-VIS spectrophotometer (Agilent Technologies). The experiments were carried out in a medium, which mimic the gastrointestinal environment, using an acidic solution of pH 2.7 as dissolution medium. During the experiment the temperature was maintained at 37 °C. Aliquots of the medium of 10 mL withdrawn at predetermined time intervals were analyzed at λ_max_ of 260 nm, the characteristic wavelength for SDZ. The drug release kinetics was evaluated with a semi-empirical Equation (2) based on Korsmeyer-Peppas model, which is applied at the initial stages (approximately 60% fractional release) [46].

(2) $$\frac{M_{t}}{M_{\infty }}=kt^{n}$$

where *M_t_*/*M*_∞_ represents the fraction of the drug released; *M_t_* and *M*_∞_ are the absolute cumulative amount of drug released at time t and at infinite time, respectively (in this case the maximum amount released in the experimental conditions used, at the plateau of the release curves; *k* is a constant incorporating the characteristics of the macromolecular drug loaded system and n is the diffusional exponent characteristic for the release mechanism).

In the equation above, a value of *n* ≈ 0.5 indicates a *Fickian* diffusion mechanism of the drug from the hydrogel network, while a value 0.5 < *n* < 1 indicates an anomalous or non-*Fickian* behavior. When *n* = 1, a case II transport mechanism is involved with zero order kinetics, while *n* > 1 indicates a special case II transport mechanism [47]. The corresponding SDZ release profiles are represented through plots of the cumulative percentage of drug released *versus* time.

#### 3.4.6. Antimicrobial Tests

The antimicrobial tests were effectuated according to standard methods SR ISO 16649-2/2007 [48]. The lyophilized ATCC cultures: *Salmonella typhymurium*14028, *Listeria monocytogenes*7644 and *Escherichia coli*25922, which were purchased from American Type Culture Collection (Rockville, MD, USA) used as microorganism strains in this study. The ATCC cultures have been reconstituted according to the requirements of specific standards such as: SR EN ISO 11133/2014 [49]; ILAC G9/2005 [50]; SR EN ISO 7218-A1/2014 [51].

The lyophilized culture was subcultured to obtain replicate reference stock cultures and further the reference stock culture was subcultured to obtain the working stock culture. From this working stock culture bacterial suspensions of 0.5 McF (measured with a densitometer) were obtained. The obtained suspensions were serial diluted to achieve concentrations of about 10^2^–10^3^ UFC/0.1 mL that were used for testing the prepared polymer samples.

ATCC culture bacteria contamination, innoculation and incubation performed for 24 h and 48 h at 44 °C, identifying target germs. Sterilization of the samples was made in autoclave at 110 °C, 0.5 bars for 20 min. Preparation of ATCC cultures was done by seeding the average pre-enrichment and incubation at 37 °C for 24 h, counting the colonies in 0.1 mL culture by selective culture medium separation, seeding of 0.1 mL bacterial culture ATCC using sterile swab samples surface.

After 24 h and 48 h using sterile tampons moistened in peptone physiological serum was collected the specimens from the test surfaces. The collected specimens were seeded on the surface of specific culture media: XLD—*Xylose Lysine Desoxycholate agar* for *Salmonella typhymurium*; ALOA—*Agar Listeria Ottaviani&Agosti* for *Listeria monocytogenes*; and VRBG—*Violet Red Bile Glucose Agar* in the case of *Escherichia coli* and the specific colonies of each bacterial species were counted.

By knowing how much the sample was diluted prior to being plated, along with the amount of the dilution used in plating, the concentration of the viable cells per milliliter in the original sample was calculated. To be comparable, the reduction ratio of the bacteria was evaluated by the following equation: $R(\%)=\frac{A-B}{A}\times 100\%$, where *R* is the percentage reduction ratio; *A* is the number of bacterial colonies from the untreated bacteria suspension (without samples to be tested); and *B* is the number of bacterial colonies from the bacteria culture treated with samples under study.

## 4. Conclusions

Chitosan was functionalized with sulfadiazine without auxiliary molecules, enhancing its bacteriostatic effect and subsequently the ability to promote wound healing. Polyelectrolyte sponges prepared from SDZ-modified CS (SCS) with sodium hyaluronate showed an increased swelling capacity and a heterogeneous porous morphology with pores highly interconnected. In particular PEC Mm sample presented a significantly slower swelling rate and a more dense/compact porosity compared with other samples, correlated with a more uniform drug distribution within the matrix and the slowest sulfadiazine release rate. The samples containing CSM and especially PEC Mm allowed a more efficient entrapment of the drug within the 3D structure followed by a subsequent slower release. The prepared PECs demonstrated their antimicrobial efficiency against *Escherichia coli*, *Listeria monocytogenes* and *Salmonella thyphymurium.*

The results obtained demonstrated that the PECs prepared from SDZ-modified chitosan represent an attractive alternative as efficient systems for prolonged drug delivery with enhanced bacteriostatic effect.
